# Coupling of the spatial distributions between sMRI and PET reveals the progression of Alzheimer’s disease

**DOI:** 10.1162/netn_a_00271

**Published:** 2023-01-01

**Authors:** Kun Zhao, Jiaji Lin, Martin Dyrba, Dong Wang, Tongtong Che, Haoyang Wu, Jingyu Wang, Yong Liu, Shuyu Li

**Affiliations:** Beijing Advanced Innovation Center for Biomedical Engineering, School of Biological Science and Medical Engineering, Beihang University, Beijing, China; Department of Neurology, Second Affiliated Hospital of Air Force Medical University, Xi’an, China; German Center for Neurodegenerative Diseases (DZNE), Rostock, Germany; School of Information Science and Engineering, Shandong Normal University, Jinan, China; School of Basic Medicine, Air Force Medical University, Xi’an, China; School of Artificial Intelligence, Beijing University of Posts and Telecommunications, Beijing, China; State Key Laboratory of Cognition Neuroscience and Learning, Beijing Normal University, Beijing, China

**Keywords:** Alzheimer’s disease, Coupling, Spatial inconsistency, Multimodal, Regional radiomics similarity network, Progression

## Abstract

Amyloid-beta (Aβ) deposition and altered brain structure are the most relevant neuroimaging biomarkers for Alzheimer’s disease (AD). However, their spatial inconsistency was always confusing and misleading. Furthermore, the relationship between this spatial inconsistency and AD progression is unclear. The current study introduced a regional radiomics similarity network (R2SN) to map structural MRI and Aβ positron emission tomography (PET) images to study their cross-modal interregional coupling. A total of 790 participants (248 normal controls, 390 mild cognitive impaired patients, and 152 AD patients) with their structural MRI and PET images were studied. The results showed that global and regional R2SN coupling significantly decreased according to the severity of cognitive decline, from mild cognitive impairment to AD dementia. The global coupling patterns are discriminative between different APOE ε4, Aβ, and Tau subgroups. R2SN coupling was probed for relationships with neuropsychiatric measures and peripheral biomarkers. Kaplan–Meier analysis showed that lower *global coupling scores* could reveal worse clinical progression of dementia. The R2SN coupling scores derived from the coupling between Aβ and atrophy over individual brain regions could reflect the specific pathway of AD progression, which would be a reliable biomarker for AD.

## Author Summary

Amyloid-beta (Aβ) deposition and altered brain structure are the most relevant neuroimaging biomarkers for Alzheimer’s disease (AD). We introduced a novel network coupling measure based on the regional radiomics similarity network (R2SN) to explore the potential association between the spatial distributions of brain structure and Aβ based on sMRI and Aβ positron emission tomography (PET) imaging. In this study, we systematically demonstrated that the alteration of the coupling between brain networks of brain structure and Aβ accumulation could serve as a predictor for revealing the distinct progression of AD.

## INTRODUCTION

Both amyloid-beta (Aβ) accumulation and altered brain structure and function are the most relevant noninvasive biomarkers for Alzheimer’s disease (AD) across the spectrum of subjective cognitive decline, mild cognitive impairment (MCI), and AD dementia (P. Chen et al., 2022; Lista et al., 2014; Rathore et al., 2017; H. Wang et al., 2021). In the classical amyloid cascade hypothesis, it is suggested that Aβ aggregation and deposition in the brain parenchyma initiate a sequence of events that further lead to neuronal death, which eventually leads to atrophy and dementia (Hardy & Higgins, 1992). The idea that higher Aβ causes faster neurodegeneration has been very influential in research (Sepulcre et al., 2018; Zhang et al., 2020). However, the temporal sequence and causal relationship between Aβ spreading and atrophy signatures have been questioned because of their spatial inconsistency during AD progression. Aβ deposition starts in association cortices and spreads from the neocortex to the allocortex, but brain structure alterations start in the hippocampus and spread from the medial temporal lobe to the frontal lobe and then progress to the whole brain (van der Kant et al., 2020; W. Y. Wang et al., 2015; Yang et al., 2012; Young et al., 2018). Recent studies indicate that neuronal injury does not necessarily occur where Aβ plaques are deposited (Terry et al., 1991). Amyloidosis-defined “pure AD neuropathology” is observed in only 30%–50% of patients with probable AD dementia based on sMRI (Robinson et al., 2018). In general, the relationship between Aβ accumulation and altered brain structure is still inconclusive. Meanwhile, finding a more accurate analysis method for their relationship has potentially valuable implications for understanding the pathogenesis of AD.

Some efforts have been made to obtain cross-modal images of Aβ accumulation and structural architecture. A previous study applied partial correlation analysis for space-normalized 18F-florbetapir positron emission tomography (PET) and T1-weighted MRI scans and found a negative relationship between global amyloid load and gray matter volume in preclinical AD cases (X. Wang et al., 2021). A similar covariance-pattern method was applied to cognitively normal elderly individuals to identify the collaborative relationship between Aβ deposition and atrophy (Oh et al., 2014). All of the previous studies have demonstrated that the different atrophy patterns driven by Aβ deposition might lead to distinct AD progression. However, spatial resolution varies within the imaging modalities, and inherently lower signal/noise ratios (such as PET) make it unlikely that the features extracted from a particular imaging modality will have the same association with the underlying characteristics as those from another imaging modality (Cook et al., 2018). Network mapping of images may be an excellent solution to this type of problem. For example, by calculating the correlation coefficient in the time series between each pair of regions of interest, functional networks were frequently applied to analyze the characteristics of brain network dynamics (Alexander-Bloch et al., 2013). Interregional similarity networks, such as structural covariance networks (SCNs) or morphometric similarity networks (MSNs), have been shown to be powerful approaches to capturing anatomical indices (Seidlitz et al., 2018). This is because the brain is a complex information transmission system (Bullmore & Sporns, 2012), and cross-regional mining based on a large-scale network is better for describing the relevant properties within the brain than is isolated regional analysis (Alexander-Bloch et al., 2013; Bullmore & Sporns, 2009; Dyrba et al., 2020; Luppi & Stamatakis, 2021; Tijms et al., 2012).

Here, we introduced a novel network coupling measure based on the regional radiomics similarity network (R2SN) (Zhao, Zheng, Che, et al., 2021; Zhao, Zheng, Dyrba, et al., 2022) to explore the potential association between the spatial distributions of Aβ and brain structure based on sMRI and Aβ PET imaging. Radiomics features can provide comprehensive and sensitive information about brain regions. Network mapping of both Aβ deposition and brain atrophy signatures based on radiomics features provides better inter- and inner-modal information on spatial distribution (Z. J. Chen et al., 2008; Z. Liu et al., 2021). It can capture alterations in the AD morphological covariation network with robustness, stability, and a biological basis and serve as a better biomarker in disease diagnosis, mechanistic studies, and progression tracking than can traditional MRI measures (Zhao, Zheng, Che, et al., 2021; Zhao, Zheng, Dyrba, et al., 2022). The R2SN coupling of sMRI and PET networks was computed to evaluate coalterations among different brain anatomical regions, which provides a new comprehensive measure for the spatial distribution of the brain rather than isolated brain regions (Alexander-Bloch et al., 2013; Zhao, Zheng, Che, et al., 2021). We evaluated the basis of this R2SN coupling and further systematic findings that may be a predictor for revealing the relationship between Aβ deposition and structural alterations.

## MATERIALS AND METHODS

### Data Acquisition and Preprocessing

This study included 790 participants (248 normal controls [NCs], 390 MCI patients, and 152 AD patients) with their T1 sMRI and Aβ PET images of AV45 tracer from the Alzheimer’s Disease Neuroimaging Initiative (ADNI, https://adni.loni.usc.edu). The clinical measures included Mini–Mental State Examination (MMSE) scores, and Rey Auditory Verbal Learning Test (AVLT; including AVLT1: Immediate, AVLT2: Learning), Alzheimer’s Disease Assessment Scale (ADAS-cog11 and ADAS-cog13), cerebrospinal fluid (CSF) Aβ, CSF Tau, CSF phosphorylated Tau (p-Tau), and fluorodeoxyglucose (FDG) PET were obtained from the ADNI. Detailed information can be found in Table 1 and Supporting Information S01. All 790 subjects have been previously reported (Ding et al., 2021; Zhao, Zheng, Dyrba, et al., 2022). The primary aim of those previous studies was to verify whether radiomics features based on PET images could serve as biomarkers for AD and whether the radiomics similarity network based on sMRI could be applied to AD and MCI subtypes. Here, the current study introduced a R2SN to map structural MRI and Aβ PET imaging in order to study their cross-modal interregional coupling.

**Table 1.** Detailed information on the subjects included in this study

	Group	Age (years)	Sex (M/F)	Clinical measure
All subjects	NC (248)	73.78 ± 6.08	125/123	/
	MCI (390)	71.93 ± 7.37	217/173	/
	AD (152)	73.82 ± 7.37	89/63	/
	*P*	0.002	0.24	/
Subjects with an MMSE	NC (247)	73.72 ± 6.02	125/122	29.08 ± 1.19
	MCI (388)	71.96 ± 7.37	217/171	28.03 ± 1.81
	AD (152)	73.82 ± 7.37	89/63	22.11 ± 3.63
	*P*	0.002	0.24	<0.001
Subjects with an FDG measurement	NC (238)	73.77 ± 6.12	120/118	1.31 ± 0.11
	MCI (383)	71.85 ± 7.38	214/169	1.25 ± 0.13
	AD (144)	73.84 ± 7.51	83/61	1.06 ± 0.15
	*P*	0.001	0.29	<0.001
Subjects with an Aβ	NC (131)	73.46 ± 6.11	65/66	1,038.42 ± 386.43
	MCI (278)	71.86 ± 7.18	157/121	888.74 ± 343.83
	AD (96)	73.79 ± 7.65	55/41	646.57 ± 282.88
	*P*	0.021	0.37	<0.001
Subjects with a Tau	NC (186)	73.24 ± 6.04	93/93	243.99 ± 95.84
	MCI (324)	71.55 ± 7.28	175/149	274.52 ± 127.33
	AD (101)	74.68 ± 7.76	60/41	379.12 ± 153.46
	*P*	0.005	0.31	<0.001
Subjects with a p-Tau	NC (185)	73.28 ± 6.03	92/93	22.59 ± 10.09
	MCI (324)	71.55 ± 7.28	175/149	26.30 ± 14.14
	AD (101)	73.66 ± 7.61	60/41	37.18 ± 16.65
	*P*	0.001	0.29	<0.001
Subjects with an ADAS-cog11	NC (248)	73.78 ± 6.08	125/123	5.85 ± 2.95
	MCI (389)	71.96 ± 7.36	216/173	9.09 ± 4.30
	AD (151)	73.76 ± 7.36	88/63	21.61 ± 8.22
	*P*	0.001	0.26	<0.001
Subjects with an ADAS-cog13	NC (246)	73.74 ± 6.05	123/123	9.22 ± 4.48
	MCI (387)	71.93 ± 7.36	215/172	14.70 ± 6.63
	AD (147)	73.72 ± 7.34	86/61	32.21 ± 9.59
	*P*	0.002	0.21	<0.001
Subjects with an AVLT1	NC (247)	73.75 ± 6.08	124/123	45.09 ± 9.87
	MCI (389)	71.90 ± 7.36	216/173	37.26 ± 11.15
	AD (150)	73.82 ± 7.24	89/61	21.37 ± 7.39
	*P*	0.001	0.18	<0.001
Subjects with an AVLT2 (*N* = 1,575)	NC (247)	73.75 ± 6.08	124/123	6.01 ± 2.32
	MCI (389)	71.90 ± 7.36	216/173	4.63 ± 2.64
	AD (150)	73.82 ± 7.24	89/61	1.51 ± 1.63
	*P*	0.001	0.18	<0.001

For each participant, the T1 MRI image was aligned with Montreal Neurological Institute (MNI) space using Advanced Normalization Tools (ANTs) after N4 bias field correction (https://github.com/ANTsX/ANTsPy). The preliminary preprocessing of the Aβ PET image was performed by the ADNI group (https://adni.loni.usc.edu/methods/pet-analysis-method/pet-analysis/#pet-pre-processing-container). The Aβ PET image was also registered to MNI standard space using the ANTs toolkit.

### R2SN Construction and Cross-Modal Coupling

**Figure 1.**
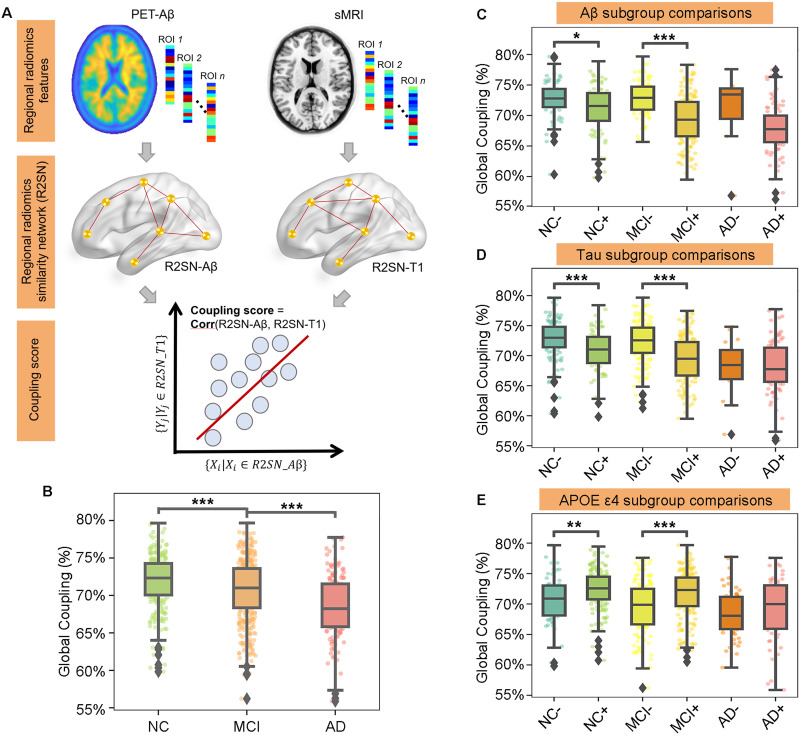
Global R2SN coupling patterns were discriminative for cognitively impaired states. (A) Overview of the study methodology. (B) The global coupling scores were significantly different among the NC, MCI, and AD groups. (C–E) Further subgroup comparisons were computed in each cognitively impaired state according to Aβ, Tau, and APOE ε4. * *p* < 0.05, ** *p* < 0.01, *** *p* < 0.001.

The entire experimental process is shown in Figure 1A. For each image, a series of radiomics features (*N* = 47) were extracted in each brain region (total of 246) defined by the Brainnetome Atlas (Fan et al., 2016). The definitions and detailed descriptions can be found in previous publications (Aerts et al., 2014; Ding et al., 2021; Zhao, Ding, et al., 2020; Zhao, Zheng, Che, et al., 2021; Zhao, Zheng, Dyrba, et al., 2022) and are listed in Supporting Information S02. All features were described by Aerts and colleagues and implemented as in-house MATLAB scripts (https://github.com/YongLiulab/; Y. Liu, 2022). A min-max method was first introduced to normalize the radiomics features among different brain regions, and the redundancy features were removed in further analysis, which was defined as those features that had a high correlation with other features (*R* > 0.9), based on previous studies (Zhao, Zheng, Che, et al., 2021; Zhao, Zheng, Dyrba, et al., 2022). The node was defined as the brain regions defined by the Brainnetome Atlas, and the edge was defined as the Pearson’s correlations between interregional radiomics features. As a result, two models of R2SN (R2SN-T1, R2SN-Aβ) were constructed for each participant. Detailed information can be found in Supporting Information S02 and S03.

To evaluate the relationship between R2SN-T1 and R2SN-Aβ, we defined the coupling of the two networks at the global and local levels. Briefly, the global coupling score was defined as the Pearson’s correlations between the edge of R2SN-T1 and R2SN-Aβ.

$$\text{Global}\;\text{coupling}\;\text{score}=\text{Corr}\left(\left\{M^{ij}|M^{ij}\in R2SN‐T1\right\},\left\{N^{ij}|N^{ij}\in R2SN‐A\beta \right\}\right).$$

Briefly, all edges of the R2SN based on MRI can be converted into a vector with 30,315 × 1 (upper triangular matrix of 246 × 246), the same as R2SN based on PET. Here, the correlations between two networks were converted into Pearson’s correlations between two vectors with 30,315 × 1.

The local coupling score was defined as the Pearson’s correlations between the connections of each node based on R2SN-T1 and R2SN-Aβ (Figure 1).

$$\text{Local}\;\text{coupling}\;{\text{score}}_{i}=\text{Corr}\left(\left\{x_{ij|j=1,\ldots ,246}\in R2SN‐T1\right\},\left\{y_{ij|j=1,\ldots ,246}\in R2SN‐A\beta \right\}\right),$$

where *i* and *j* represent the *i*th and *j*th brain regions, respectively.

### Grouped Comparison for Cognitively Impaired States

We first tested whether the global coupling score was differentiable among the NC, MCI, and AD groups by ANOVA with age and sex as the covariates. Then, a two-tailed, two-sample *t* test was used to estimate the difference in the global coupling score in AD versus NC, MCI versus NC, and AD versus MCI, with age and sex as the covariates. Then, further subgroup comparisons were computed according to apolipoprotein E (APOE) ε4, Aβ, and Tau. Aβ+ was defined as a CSF Aβ value < 980 pg/ml, and Tau+ was defined as a CSF total Tau > 245 pg/ml, based on https://files.alz.washington.edu/presentations/2018/spring/biomarkers/SHAW.pdf and a previous study (Zhao, Zheng, Dyrba, et al., 2022). The local coupling scores were analyzed among the NC, MCI, and AD groups with a two-tailed, two-sample *t* test with age and sex as the covariates.

### Clinical Significance of the Global Coupling Score

To investigate the potential biological basis of the global coupling score, Pearson correlations between the global coupling score and neuropsychiatric measures (including MMSE, AVLT1, AVLT2, ADAS-cog11, and ADAS-cog13) and peripheral biomarkers (including CSF Aβ, CSF Tau, CSF p-Tau, and FDG) were computed with age and sex as covariates, as in previous studies (Ding et al., 2021; Zhao, Zheng, Dyrba, et al., 2022). To further assess whether the coupling level between R2SN-T1 and R2SN-Aβ can reveal the different clinical progression, the MCI patients were subdivided into two subgroups (0%–50% with low coupling scores and 50%–100% with high coupling scores) and four subgroups (S1: 0%–25%, S2: 25%–50%, S3: 50%–75%, and S4: 75%–100%). Survival curves for each subgroup were computed with Kaplan–Meier analysis based on real follow-up duration information. Here, 1 was defined as the MCI subject developing to AD, and 0 was defined as the MCI subject not developing to AD (Li et al., 2019).

## RESULTS

### Demographic and Neuropsychological Characteristics

A total of 790 participants were studied, including 248 NCs, 390 MCI patients, and 152 AD patients. The mean age was significantly different (*P* = 0.002) among the groups, and the sex proportion was not significantly different (*P* = 0.24). The clinical measures (including MMSE score, ADAS-cog11 score, ADAS-cog13 score, CSF Aβ level, CSF Tau level, CSF p-Tau level, AVLT score, and FDG) were significantly different among the NC, MCI, and AD groups (*P* < 0.001 with ANOVA; Table 1).

### Global R2SN Coupling Patterns Are Discriminative for Cognitively Impaired States

The global coupling score for NCs was 0.72 ± 0.04, while the global coupling scores for MCI and AD patients were 0.71 ± 0.04 and 0.68 ± 0.04, respectively (Figure 1B). The global coupling scores were significantly different among the NC, MCI, and AD groups (*P* < 0.001 by ANOVA), with scores of AD significantly lower than those of MCI (*P* < 0.001 by *t* test), scores of AD significantly lower than those of NC (*P* < 0.001 by *t* test), and scores of MCI significantly lower than those of NC (*P* < 0.001 by *t* test; Figure 1B).

Further subgroup comparisons were computed in each cognitively impaired state according to Aβ, Tau, and APOE ε4. The global coupling scores were significantly decreased in the Aβ+ NC subgroup compared with the Aβ− subgroup (*P* = 0.015) and decreased in the Aβ+ MCI subgroup compared with the Aβ− MCI subgroup (*P* < 0.001; Figure 1C). They were also significantly decreased in the Tau+ NC subgroup compared with the Tau− NC subgroup (*P* < 0.001) and decreased in the Tau+ MCI subgroup compared with the Tau− MCI subgroup (*P* < 0.001; Figure 1D). There was also a significant decrease in global coupling scores in APOE ε4+ NC and MCI in contrast with APOE ε4− NC and MCI, respectively (*P* = 0.003 and *P* < 0.001; Figure 1E).

### Global Coupling Scores Were Significantly Correlated With Clinical Measures

**Figure 2.**
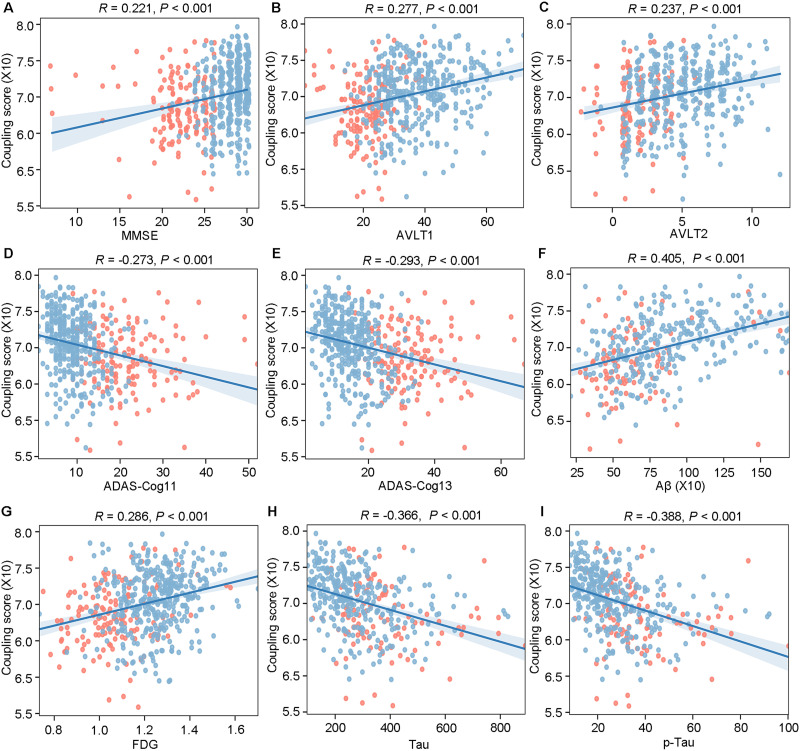
Global coupling scores were significantly correlated with clinical measures. The correlation between global coupling score and (A) MMSE, (B) AVLT1, (C) AVLT2, (D) ADAS-Cog11, (E) ADAS-Cog13, (F) Aβ, (G) FDG, (H) Tau, and (I) p-Tau. The blue dots indicate the MCI subjects, and the red dots indicate the AD subjects.

Multiple clinical measures were positively correlated with the global coupling scores, including MMSE (*R* = 0.221, *P* < 0.001), AVLT1 (*R* = 0.277, *P* < 0.001), and AVLT2 (*R* = 0.237, *P* < 0.001; Figure 2A–C), while ADAS-cog11 (*R* = −0.273, *P* < 0.001) and ADAS-cog13 (*R* = −0.293, *P* < 0.001) were found to be negatively correlated with the global coupling scores (Figure 2D–E). Meanwhile, there were also positive correlations between the global coupling scores and CSF Aβ (*R* = 0.405, *P* < 0.001) and FDG (*R* = 0.286, *P* < 0.001; Figure 2F–G), as well as negative correlations with Tau (*R* = −0.366, *P* < 0.001) and p-Tau (*R* = −0.388, *P* < 0.001; Figure 2H–I), all of which were corrected by Bonferroni correction with *P* < 0.05/9.

### Local Coupling Scores Suggested Regional Heterogeneity Links to Aβ and Atrophy

**Figure 3.**
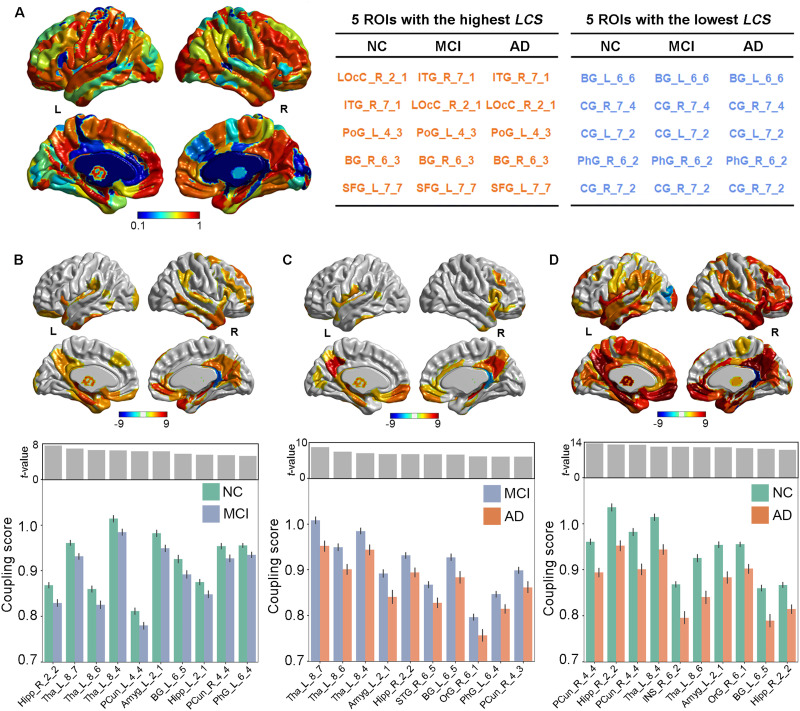
Local coupling scores suggested regional heterogeneity in the link between Aβ and atrophy. (A) Overview of the general local coupling scores across the NC, MCI, and AD groups as well as their representative brain regions. The color bar represents the strength of local coupling scores. The discriminative regions of local R2SN coupling patterns in (B) MCI vs. NC, (C) AD vs. MCI and (D) AD vs. NC. The color bar represents the *T* scores between each pair of groups. The bottom bar figures indicate the top 10 regions between each pair of groups. The error bar indicates the standard deviation of the coupling score. LCS = local coupling scores.

After clarifying the global R2SN coupling patterns, we used the image datasets to analyze regional heterogeneity. Overall, in the AD, MCI, and NC groups, the distribution of regional R2SN coupling was basically the same, with LOcC_R_2_1, ITG_R_7_1, PoG_L_4_3, BG_R_6_3, and SFG_L_7_7 having the highest scores and BG_L_6_6, CG_R_7_4, CG_L_7_2, PhG_R_6_2, and CG_R_7_2 having the lowest scores (Figure 3A). The detailed names of the brain regions are shown in Table S5 in the Supporting Information.

We further compared regional R2SN coupling across cognitively impaired state groups with age and sex as covariates, and there was a gradual decrease from NC to MCI/AD. The local coupling scores were significantly decreased in the MCI group compared with the NC group, including PCun_L_4_4 (*t* = 7.54, *P* < 0.001), Hipp_R_2_2 (*t* = 6.90, *P* < 0.001), PCun_R_4_4 (*t* = 6.58, *P* < 0.001), Tha_L_8_4 (*t* = 6.51, *P* < 0.001), and INS_R_6_2 (*t* = 6.32, *P* < 0.001; Figure 3B). The local coupling scores of AD were also decreased compared with MCI, including Tha_L_8_7 (*t* = 8.66, *P* < 0.001), Tha_L_8_6 (*t* = 7.44, *P* < 0.001), Tha_L_8_4 (*t* = 7.03, *P* < 0.001), Amg_L_2_1 (*t* = 6.77, *P* < 0.001), and Hipp_R_2_2 (*t* = 6.76, *P* < 0.001; Figure 3C). Finally, there was a dramatic decrease in local coupling scores in AD compared with NC, such as Hipp_R_2_2 (*t* = 13.39, *P* < 0.001), Tha_L_8_7 (*t* = 12.98, *P* < 0.001), Tha_L_8_6 (*t* = 12.87, *P* < 0.001), Tha_L_8_4 (*t* = 12.13, *P* < 0.001), and PCun_L_4_4 (*t* = 12.09, *P* < 0.001; Figure 3D). It should be noted that all of the brain regions shown in Figure 3 have been corrected by Bonferroni correction with *P* < 0.05/246. Detailed information on these regions can be found at https://atlas.brainnetome.org/index.html.

### Global Coupling Scores Indicate the Distinct Progression of MCI Patients

**Figure 4.**
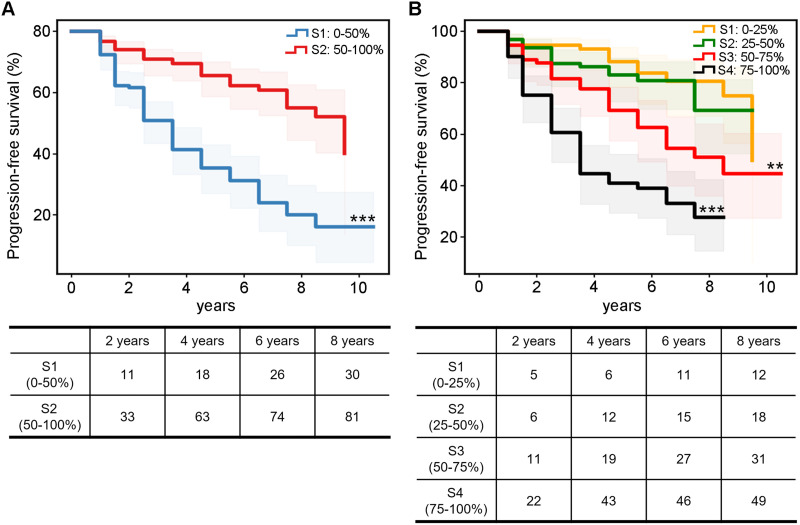
Global coupling scores indicated the distinct progression of the MCI patients. The survival curve of the different coupling levels: (A) two equal subgroupings and (B) four quartile subgroupings. ** *p* < 0.01, *** *p* < 0.001.

To further assess whether the R2SN coupling level could reveal the clinical progression of dementia, we subdivided the MCI patients into two subgroups according to the average global coupling scores. Kaplan–Meier analysis demonstrated that the progression pattern of the low-coupling subgroup was significantly worse than that of the high-coupling subgroup (*P* < 0.001; Figure 4A). To further confirm this result, we subdivided the MCI patients into four subgroups according to the quartiles of global coupling scores (S1: top 0%–25%, S2: 25%–50%, S3: 50%–75%, and S4: 75%–100%). Kaplan–Meier analysis showed that lower global coupling scores could reveal worse clinical progression of dementia (*P*_S1 vs. S2_ = 0.466, *P*_S1 vs. S3_ = 0.002, *P*_S1 vs. S4_ < 0.001, *P*_S2 vs. S3_ = 0.014, *P*_S2 vs. S4_ < 0.001, *P*_S3 vs. S4_ < 0.001; Figure 4B).

## DISCUSSION

The initial motivation for establishing these network mappings is to use analytical tools from graph theory, system theory, control theory, and the like to mine the underlying peculiarities behind the original data. There are also many times when networking data can lead to unique mechanistic annotations; for example, human MSNs recapitulate cortical cytoarchitectonic divisions and better structural connectomes (Seidlitz et al., 2018). In the present study, we applied R2SNs to construct a morphological covariation network by radiomics, providing a network perspective to analyze the relationship between Aβ accumulation and altered brain structure.

Network relationships can be investigated using methods ranging from simple approaches, such as statistical models (Messé et al., 2014; Mišić et al., 2016), to more complex ones, such as communication models (Goñi et al., 2014; Mišić et al., 2015) or biophysical models (Breakspear, 2017; Sanz-Leon et al., 2015). Typically, network coupling by correlational analyses is simple but useful, and it could offer new insights into individual fingerprinting, that is, how functional brain networks align with the underlying structural network as measured with diffusion MRI (Honey et al., 2009) or how microstructural covariance network is correlated with region-to-region connectivity (Huntenburg et al., 2017; Paquola et al., 2019). This statistical model offers a data-driven way to associate structural and functional connectivity without assuming a specific mode of interaction among neuronal populations, which has proven to be helpful in quantifying the effects of manipulations and perturbations, such as development and aging (Baum et al., 2020), neurological and psychiatric diseases (Jirsa et al., 2017), and lesions (Rosenthal et al., 2018). Extended studies further explain this coupling as systematic hierarchical variation in laminar differentiation (Paquola et al., 2019) and cytoarchitecture (Vazquez-Rodriguez et al., 2019). Similar to the causal interpretation between brain structure and function, brain atrophy was always taken as an inevitable event associated with Aβ deposition in dementia progression (Sepulcre et al., 2018; Zhang et al., 2020). Several AD studies have shown a temporal sequence between the spreading of Aβ aggregation species and other neurodegeneration-based biological signatures underlying atrophy, including tauopathy, neuroinflammation, and neurochemical systems (Hampel et al., 2021). Although no causal effect was established between the Aβ pathway and AD-related pathophysiological changes, it has been suggested that the Aβ pathway exerts a permissive/facilitating effect on other pathophysiological pathways and/or unfolds synergistically at different temporal scales (Hampel et al., 2021). The relationship between the R2SN-T1 and R2SN-Aβ networks could better reflect the complex process secondary to the Aβ pathway and its direct link to atrophy.

In the analysis of local coupling scores, it was found that the spatial coupling distribution of brain regions of both the NC and clinical groups was very similar, with the highest local coupling scores in LOcC_R_2_1, ITG_R_7_1, PoG_L_4_3, BG_R_6_3, and SFG_L_7_6 and the lowest local coupling scores in BG_L_6_6, CG_R_7_4, CG_L_7_2, PhG_R_6_2, and CG_r_7_2. The consistent distribution of local coupling scores across different groups once again suggested that the contribution of Aβ to the disease varied; that is, the contribution to the morphological changes of brain tissue was higher in brain regions such as the temporal lobe. Future biological studies of highly coupled areas may provide a more in-depth mechanistic explanation.

In further analysis of global and local coupling scores, we noticed that the coupling pattern constantly declines with the severity of cognitive impairment. A previous study highlighted that the coupling of altered gray matter volume and Aβ was correlated with subjective cognitive decline–related worries (X. Wang et al., 2021). Even in cognitively normal elderly individuals, there was a covarying alteration in Aβ deposition and atrophy (Oh et al., 2014). However, the contribution of Aβ to brain atrophy gradually decreased. In the early course of cognitively impaired states rather than normal aging, Aβ depositions acted as a more pronounced accelerator to advance the onset of brain degeneration (Donohue et al., 2017; Fandos et al., 2017; Lim et al., 2014; Sheline et al., 2010). Aβ deposition of upstream events drives downstream events (neocortical Tau spread, impaired glucose metabolism, and widespread neurodegeneration; Hansson, 2021). These inconsistent biomarkers also result from the different AD pathways with distinct progression (Reimand et al., 2020). This significant stage effect leads to the differential sensitivity of classical ATN (A [CSF Ab_42_, or Aβ_42_/Ab_40_ ratio and amyloid PET], T [CSF p-Tau, Tau PET], and N [atomic MRI, FDG PET, and CSF total Tau]) in the diagnosis sensitivity over AD progression (Hammond et al., 2020; Sperling et al., 2011). We supposed that the lower the coupling, the more abnormal pathophysiological mechanisms were involved, which was also an important reason for the poor prognosis of AD. This hypothesis is also reflected in the progressive disease outcome of MCI patients. The lower the global coupling scores are, the faster the rate at which MCI patients transition to AD. Brain regions with lower local coupling scores tended to be brain regions with more obvious atrophy changes in previous reports (Grothe et al., 2018). All these results indicate that the progression of AD is the result of the simultaneous effect of multiple biological mechanisms, and the assessment of the contribution of different biomarkers may have clinical value in predicting disease prognosis.

This study has some limitations. First, the sMRI and PET scans for some subjects were not obtained at the same time (e.g., 3–6 months). Second, we explored the coupling score of the R2SN between sMRI and PET in only the ADNI dataset. Another dataset to verify the robustness should be analyzed in future studies. Third, the altered coupling pattern among different imaging modalities would benefit our understanding of AD pathophysiology in the future.

### Conclusion

In this study, we systematically demonstrated that the alteration of the coupling between brain networks of brain structure and Aβ accumulation was related to the solid biological/clinical basis, which could serve as a predictor for revealing the distinct progression of AD. This study provided a new measure for exploring the coupling between Aβ and atrophy.

## ACKNOWLEDGMENTS

Data collection and sharing for this project were funded by the Alzheimer’s Disease Neuroimaging Initiative (ADNI) (National Institutes of Health Grant No. U01 AG024904) and DOD ADNI (Department of Defense Award No. W81XWH-12-2-0012). The ADNI was funded by the National Institute on Aging, the National Institute of Biomedical Imaging and Bioengineering, and generous contributions from AbbVie; Alzheimer’s Association; Alzheimer’s Drug Discovery Foundation; Araclon Biotech; BioClinica, Inc.; Biogen; Bristol-Myers Squibb Company; CereSpir, Inc.; Cogstate; Eisai Inc.; Elan Pharmaceuticals, Inc.; Eli Lilly and Company; EuroImmun; F. Hoffmann-La Roche Ltd., and its affiliated company Genentech, Inc.; Fujirebio; GE Healthcare; IXICO, Ltd.; Janssen Alzheimer Immunotherapy Research & Development, LLC.; Johnson & Johnson Pharmaceutical Research & Development, LLC; Lumosity; Lundbeck; Merck & Co., Inc.; Meso Scale Diagnostics, LLC.; NeuroRx Research; Neurotrack Technologies; Novartis Pharmaceuticals Corporation; Pfizer, Inc.; Piramal Imaging; Servier; Takeda Pharmaceutical Company; and Transition Therapeutics. The Canadian Institutes of Health Research provided funds to support ADNI clinical sites in Canada. Private sector contributions were facilitated by the Foundation for the National Institutes of Health (https://www.fnih.org). The grantee organization was the Northern California Institute for Research and Education, and the study was coordinated by the Alzheimer’s Therapeutic Research Institute at the University of Southern California. ADNI data were disseminated by the Laboratory of Neuro Imaging at the University of Southern California. As data used in preparation of this article were obtained from the ADNI database (https://adni.loni.usc.edu), the investigators within the ADNI contributed to the design and implementation of the ADNI and/or provided data but did not participate in the analysis or writing of this report. A complete listing of ADNI investigators can be found at https://adni.loni.usc.edu/wp-content/uploads/how_to_apply/ADNI_Acknowledgement_List.pdf.

## DATA AVAILABILITY

All subjects of this study were downloaded from the ADNI (https://adni.loni.usc.edu), and the scripts of the radiomics features (Zhao, Ding, et al., 2020) are available at https://github.com/YongLiulab (Y. Liu, 2022).

## SUPPORTING INFORMATION

Supporting information for this article is available at https://doi.org/10.1162/netn_a_00271.

## AUTHOR CONTRIBUTIONS

Kun Zhao: Conceptualization; Data curation; Formal analysis; Investigation; Methodology; Writing – original draft. Jiaji Lin: Conceptualization; Investigation; Writing – original draft. Martin Dyrba: Writing – review & editing. Dong Wang: Data curation. Tongtong Che: Writing – review & editing. Haoyang Wu: Software. Jingyu Wang: Software. Yong Liu: Conceptualization; Supervision; Validation; Visualization; Writing – review & editing. Shuyu Li: Conceptualization; Supervision; Validation; Writing – review & editing.

## FUNDING INFORMATION

Shuyu Li, Startup Funds for Leading Talents at Beijing Normal University. Shuyu Li, National Natural Science Foundation of China, Award ID: 81972160. Yong Liu, Fundamental Research Funds for the Central Universities, Award ID: 2021XD-A03-1. Yong Liu, Beijing Natural Science Funds for Distinguished Young Scholars, Award ID: JQ20036. Yong Liu, National Natural Science Foundation of China, Award ID: 81871438. Yong Liu, National Natural Science Foundation of China, Award ID: 82172018.

## TECHNICAL TERMS

Regional radiomics similarity network (R2SN): A novel brain network from sMRI based on the similarity of regional radiomics features. It has been used successfully in investigating the individual cognitive and defining the subtypes of mild cognitive impairment.
Radiomics: A powerful, robust method to extract more detailed information from each brain region.

## Supporting Information

https://github.com/YongLiulab

## References

[bib1] Aerts, H. J., Velazquez, E. R., Leijenaar, R. T., Parmar, C., Grossmann, P., Carvalho, S., Bussink, J., Monshouwer, R., Haibe-Kains, B., Rietveld, D., Hoebers, F., Rietbergen, M. M., Leemans, C. R., Dekker, A., Quackenbush, J., Gillies, R. J., & Lambin, P. (2014). Decoding tumour phenotype by noninvasive imaging using a quantitative radiomics approach. Nature Communications, 5, 4006. 10.1038/ncomms5006, 24892406 PMC4059926

[bib2] Alexander-Bloch, A., Giedd, J. N., & Bullmore, E. (2013). Imaging structural co-variance between human brain regions. Nature Reviews Neuroscience, 14(5), 322–336. 10.1038/nrn3465, 23531697 PMC4043276

[bib3] Baum, G. L., Cui, Z., Roalf, D. R., Ciric, R., Betzel, R. F., Larsen, B., Cieslak, M., Cook, P. A., Xia, C. H., Moore, T. M., Ruparel, K., Oathes, D. J., Alexander-Bloch, A. F., Shinohara, R. T., Raznahan, A., Gur, R. E., Gur, R. C., Bassett, D. S., & Satterthwaite, T. D. (2020). Development of structure-function coupling in human brain networks during youth. Proceedings of the National Academy of Sciences, 117(1), 771–778. 10.1073/pnas.1912034117, 31874926 PMC6955327

[bib4] Breakspear, M. (2017). Dynamic models of large-scale brain activity. Nature Neuroscience, 20(3), 340–352. 10.1038/nn.4497, 28230845

[bib5] Bullmore, E., & Sporns, O. (2009). Complex brain networks: Graph theoretical analysis of structural and functional systems. Nature Reviews Neuroscience, 10(3), 186–198. 10.1038/nrn2575, 19190637

[bib6] Bullmore, E., & Sporns, O. (2012). The economy of brain network organization. Nature Reviews Neuroscience, 13(5), 336–349. 10.1038/nrn3214, 22498897

[bib7] Chen, P., Yao, H., Tijms, B. M., Wang, P., Wang, D., Song, C., Yang, H., Zhang, Z., Zhao, K., Qu, Y., Kang, X., Du, K., Fan, L., Han, T., Yu, C., Zhang, X., Jiang, T., Zhou, Y., Lu, J., … Liu, Y. (2022). Four distinct subtypes of Alzheimer’s disease based on resting-state connectivity biomarkers. Biological Psychiatry. 10.1016/j.biopsych.2022.06.019, 36137824

[bib8] Chen, Z. J., He, Y., Rosa-Neto, P., Germann, J., & Evans, A. C. (2008). Revealing modular architecture of human brain structural networks by using cortical thickness from MRI. Cerebral Cortex, 18(10), 2374–2381. 10.1093/cercor/bhn003, 18267952 PMC2733312

[bib9] Cook, G. J. R., Azad, G., Owczarczyk, K., Siddique, M., & Goh, V. (2018). Challenges and promises of PET radiomics. International Journal of Radiation Oncology, Biology, Physics, 102(4), 1083–1089. 10.1016/j.ijrobp.2017.12.268, 29395627 PMC6278749

[bib10] Ding, Y., Zhao, K., Che, T., Du, K., Sun, H., Liu, S., Zheng, Y., Li, S., Liu, B., Liu, Y., & Alzheimer’s Disease Neuroimaging Initiative. (2021). Quantitative radiomic features as new biomarkers for Alzheimer’s disease: An amyloid PET study. Cerebral Cortex, 31(8), 3950–3961. 10.1093/cercor/bhab061, 33884402

[bib11] Donohue, M. C., Sperling, R. A., Petersen, R., Sun, C. K., Weiner, M. W., Aisen, P. S., & Alzheimer’s Disease Neuroimaging Initiative. (2017). Association between elevated brain amyloid and subsequent cognitive decline among cognitively normal persons. JAMA, 317(22), 2305–2316. 10.1001/jama.2017.6669, 28609533 PMC5736301

[bib12] Dyrba, M., Mohammadi, R., Grothe, M. J., Kirste, T., & Teipel, S. J. (2020). Gaussian graphical models reveal inter-modal and inter-regional conditional dependencies of brain alterations in Alzheimer’s disease. Frontiers in Aging Neuroscience, 12, 99. 10.3389/fnagi.2020.00099, 32372944 PMC7186311

[bib13] Fan, L., Li, H., Zhuo, J., Zhang, Y., Wang, J., Chen, L., Yang, Z., Chu, C., Xie, S., Laird, A. R., Fox, P. T., Eickhoff, S. B., Yu, C., & Jiang, T. (2016). The Human Brainnetome Atlas: A new brain atlas based on connectional architecture. Cerebral Cortex, 26(8), 3508–3526. 10.1093/cercor/bhw157, 27230218 PMC4961028

[bib14] Fandos, N., Perez-Grijalba, V., Pesini, P., Olmos, S., Bossa, M., Villemagne, V. L., Doecke, J., Fowler, C., Masters, C. L., Sarasa, M., & AIBL Research Group. (2017). Plasma amyloid beta 42/40 ratios as biomarkers for amyloid beta cerebral deposition in cognitively normal individuals. Alzheimer’s and Dementia: Diagnosis, Assessment and Disease Monitoring, 8, 179–187. 10.1016/j.dadm.2017.07.004, 28948206 PMC5602863

[bib15] Goñi, J., van den Heuvel, M. P., Avena-Koenigsberger, A., Velez de Mendizabal, N., Betzel, R. F., Griffa, A., Hagmann, P., Corominas-Murtra, B., Thiran, J. P., & Sporns, O. (2014). Resting-brain functional connectivity predicted by analytic measures of network communication. Proceedings of the National Academy of Sciences, 111(2), 833–838. 10.1073/pnas.1315529111, 24379387 PMC3896172

[bib16] Grothe, M. J., Sepulcre, J., Gonzalez-Escamilla, G., Jelistratova, I., Schöll, M., Hansson, O., & Teipel, S. J. (2018). Molecular properties underlying regional vulnerability to Alzheimer’s disease pathology. Brain, 141(9), 2755–2771. 10.1093/brain/awy189, 30016411 PMC6113636

[bib17] Hammond, T. C., Xing, X., Wang, C., Ma, D., Nho, K., Crane, P. K., Elahi, F., Ziegler, D. A., Liang, G., Cheng, Q., Yanckello, L. M., Jacobs, N., & Lin, A. L. (2020). Beta-amyloid and tau drive early Alzheimer’s disease decline while glucose hypometabolism drives late decline. Communications Biology, 3(1), 352. 10.1038/s42003-020-1079-x, 32632135 PMC7338410

[bib18] Hampel, H., Hardy, J., Blennow, K., Chen, C., Perry, G., Kim, S. H., Villemagne, V. L., Aisen, P., Vendruscolo, M., Iwatsubo, T., Masters, C. L., Cho, M., Lannfelt, L., Cummings, J. L., & Vergallo, A. (2021). The amyloid-beta pathway in Alzheimer’s disease. Molecular Psychiatry, 26(10), 5481–5503. 10.1038/s41380-021-01249-0, 34456336 PMC8758495

[bib19] Hansson, O. (2021). Biomarkers for neurodegenerative diseases. Nature Medicine, 27(6), 954–963. 10.1038/s41591-021-01382-x, 34083813

[bib20] Hardy, J. A., & Higgins, G. A. (1992). Alzheimer’s disease: The amyloid cascade hypothesis. Science, 256(5054), 184–185. 10.1126/science.1566067, 1566067

[bib21] Honey, C. J., Sporns, O., Cammoun, L., Gigandet, X., Thiran, J. P., Meuli, R., & Hagmann, P. (2009). Predicting human resting-state functional connectivity from structural connectivity. Proceedings of the National Academy of Sciences, 106(6), 2035–2040. 10.1073/pnas.0811168106, 19188601 PMC2634800

[bib22] Huntenburg, J. M., Bazin, P. L., Goulas, A., Tardif, C. L., Villringer, A., & Margulies, D. S. (2017). A systematic relationship between functional connectivity and intracortical myelin in the human cerebral cortex. Cerebral Cortex, 27(2), 981–997. 10.1093/cercor/bhx030, 28184415 PMC5390400

[bib23] Jirsa, V. K., Proix, T., Perdikis, D., Woodman, M. M., Wang, H., Gonzalez-Martinez, J., Bernard, C., Bénar, C., Guye, M., Chauvel, P., & Bartolomei, F. (2017). The virtual epileptic patient: Individualized whole-brain models of epilepsy spread. NeuroImage, 145(Pt. B), 377–388. 10.1016/j.neuroimage.2016.04.049, 27477535

[bib24] Li, H., Habes, M., Wolk, D. A., & Fan, Y., for the Alzheimer’s Disease Neuroimaging Initiative and the Australian Imaging Biomarkers and Lifestyle Study of Aging. (2019). A deep learning model for early prediction of Alzheimer’s disease dementia based on hippocampal magnetic resonance imaging data. Alzheimer’s and Dementia, 15(8), 1059–1070. 10.1016/j.jalz.2019.02.007, 31201098 PMC6719787

[bib25] Lim, H. K., Nebes, R., Snitz, B., Cohen, A., Mathis, C., Price, J., Weissfeld, L., Klunk, W., & Aizenstein, H. J. (2014). Regional amyloid burden and intrinsic connectivity networks in cognitively normal elderly subjects. Brain, 137(Pt. 12), 3327–3338. 10.1093/brain/awu271, 25266592 PMC4240287

[bib26] Lista, S., Garaci, F. G., Ewers, M., Teipel, S., Zetterberg, H., Blennow, K., & Hampel, H. (2014). CSF Abeta1-42 combined with neuroimaging biomarkers in the early detection, diagnosis and prediction of Alzheimer’s disease. Alzheimer’s and Dementia, 10(3), 381–392. 10.1016/j.jalz.2013.04.506, 23850330

[bib27] Liu, Y. (2022). Yong Liu Lab, GitHub. https://github.com/YongLiulab

[bib28] Liu, Z., Palaniyappan, L., Wu, X., Zhang, K., Du, J., Zhao, Q., Xie, C., Tang, Y., Su, W., Wei, Y., Xue, K., Han, S., Tsai, S. J., Lin, C. P., Cheng, J., Li, C., Wang, J., Sahakian, B. J., Robbins, T. W., … Feng, J. (2021). Resolving heterogeneity in schizophrenia through a novel systems approach to brain structure: Individualized structural covariance network analysis. Molecular Psychiatry, 26, 7719–7731. 10.1038/s41380-021-01229-4, 34316005

[bib29] Luppi, A. I., & Stamatakis, E. A. (2021). Combining network topology and information theory to construct representative brain networks. Network Neuroscience, 5(1), 96–124. 10.1162/netn_a_00170, 33688608 PMC7935031

[bib30] Messé, A., Rudrauf, D., Benali, H., & Marrelec, G. (2014). Relating structure and function in the human brain: Relative contributions of anatomy, stationary dynamics, and non-stationarities. PLoS Computational Biology, 10(3), e1003530. 10.1371/journal.pcbi.1003530, 24651524 PMC3961181

[bib31] Mišić, B., Betzel, R. F., de Reus, M. A., van den Heuvel, M. P., Berman, M. G., McIntosh, A. R., & Sporns, O. (2016). Network-level structure-function relationships in human neocortex. Cerebral Cortex, 26(7), 3285–3296. 10.1093/cercor/bhw089, 27102654 PMC4898678

[bib32] Mišić, B., Betzel, R. F., Nematzadeh, A., Goñi, J., Griffa, A., Hagmann, P., Flammini, A., Ahn, Y. Y., & Sporns, O. (2015). Cooperative and competitive spreading dynamics on the human connectome. Neuron, 86(6), 1518–1529. 10.1016/j.neuron.2015.05.035, 26087168

[bib33] Oh, H., Habeck, C., Madison, C., & Jagust, W. (2014). Covarying alterations in Aβ deposition, glucose metabolism, and gray matter volume in cognitively normal elderly. Human Brain Mapping, 35(1), 297–308. 10.1002/hbm.22173, 22965806 PMC3600112

[bib34] Paquola, C., Vos De Wael, R., Wagstyl, K., Bethlehem, R. A. I., Hong, S. J., Seidlitz, J., Bullmore, E. T., Evans, A. C., Mišić, B., Margulies, D. S., Smallwood, J., & Bernhardt, B. C. (2019). Microstructural and functional gradients are increasingly dissociated in transmodal cortices. PLoS Biology, 17(5), e3000284. 10.1371/journal.pbio.3000284, 31107870 PMC6544318

[bib35] Rathore, S., Habes, M., Iftikhar, M. A., Shacklett, A., & Davatzikos, C. (2017). A review on neuroimaging-based classification studies and associated feature extraction methods for Alzheimer’s disease and its prodromal stages. NeuroImage, 155, 530–548. 10.1016/j.neuroimage.2017.03.057, 28414186 PMC5511557

[bib36] Reimand, J., Collij, L., Scheltens, P., Bouwman, F., Ossenkoppele, R., & Alzheimer’s Disease Neuroimaging Initiative. (2020). Association of amyloid-beta CSF/PET discordance and tau load 5 years later. Neurology, 95(19), e2648–e2657. 10.1212/WNL.0000000000010739, 32913020 PMC7963352

[bib37] Robinson, J. L., Lee, E. B., Xie, S. X., Rennert, L., Suh, E., Bredenberg, C., Caswell, C., Van Deerlin, V. M., Yan, N., Yousef, A., Hurtig, H. I., Siderowf, A., Grossman, M., McMillan, C. T., Miller, B., Duda, J. E., Irwin, D. J., Wolk, D., Elman, L., … Trojanowski, J. Q. (2018). Neurodegenerative disease concomitant proteinopathies are prevalent, age-related and APOE4-associated. Brain, 141(7), 2181–2193. 10.1093/brain/awy146, 29878075 PMC6022546

[bib38] Rosenthal, G., Vasa, F., Griffa, A., Hagmann, P., Amico, E., Goñi, J., Avidan, G., & Sporns, O. (2018). Mapping higher-order relations between brain structure and function with embedded vector representations of connectomes. Nature Communications, 9(1), 2178. 10.1038/s41467-018-04614-w, 29872218 PMC5988787

[bib39] Sanz-Leon, P., Knock, S. A., Spiegler, A., & Jirsa, V. K. (2015). Mathematical framework for large-scale brain network modeling in The Virtual Brain. NeuroImage, 111, 385–430. 10.1016/j.neuroimage.2015.01.002, 25592995

[bib40] Seidlitz, J., Váša, F., Shinn, M., Romero-Garcia, R., Whitaker, K. J., Vértes, P. E., Wagstyl, K., Kirkpatrick Reardon, P., Clasen, L., Liu, S., Messinger, A., Leopold, D. A., Fonagy, P., Dolan, R. J., Jones, P. B., Goodyer, I. M., Raznahan, A., & Bullmore, E. T. (2018). Morphometric similarity networks detect microscale cortical organization and predict inter-individual cognitive variation. Neuron, 97(1), 231–247. 10.1016/j.neuron.2017.11.039, 29276055 PMC5763517

[bib41] Sepulcre, J., Grothe, M. J., d’Oleire Uquillas, F., Ortiz-Teran, L., Diez, I., Yang, H. S., Jacobs, H. I. L., Hanseeuw, B. J., Li, Q., El-Fakhri, G., Sperling, R. A., & Johnson, K. A. (2018). Neurogenetic contributions to amyloid beta and tau spreading in the human cortex. Nature Medicine, 24(12), 1910–1918. 10.1038/s41591-018-0206-4, 30374196 PMC6518398

[bib42] Sheline, Y. I., Raichle, M. E., Snyder, A. Z., Morris, J. C., Head, D., Wang, S., & Mintun, M. A. (2010). Amyloid plaques disrupt resting state default mode network connectivity in cognitively normal elderly. Biological Psychiatry, 67(6), 584–587. 10.1016/j.biopsych.2009.08.024, 19833321 PMC2829379

[bib43] Sperling, R. A., Aisen, P. S., Beckett, L. A., Bennett, D. A., Craft, S., Fagan, A. M., Iwatsubo, T., Jack, C. R., Jr., Kaye, J., Montine, T. J., Park, D. C., Reiman, E. M., Rowe, C. C., Siemers, E., Stern, Y., Yaffe, K., Carrillo, M. C., Thies, B., Morrison-Bogorad, M., … Phelps, C. H. (2011). Toward defining the preclinical stages of Alzheimer’s disease: Recommendations from the National Institute on Aging–Alzheimer’s Association workgroups on diagnostic guidelines for Alzheimer’s disease. Alzheimer’s and Dementia, 7(3), 280–292. 10.1016/j.jalz.2011.03.003, 21514248 PMC3220946

[bib44] Terry, R. D., Masliah, E., Salmon, D. P., Butters, N., DeTeresa, R., Hill, R., Hansen, L. A., & Katzman, R. (1991). Physical basis of cognitive alterations in Alzheimer’s disease: Synapse loss is the major correlate of cognitive impairment. Annals of Neurology, 30(4), 572–580. 10.1002/ana.410300410, 1789684

[bib45] Tijms, B. M., Series, P., Willshaw, D. J., & Lawrie, S. M. (2012). Similarity-based extraction of individual networks from gray matter MRI scans. Cerebral Cortex, 22(7), 1530–1541. 10.1093/cercor/bhr221, 21878484

[bib46] van der Kant, R., Goldstein, L. S. B., & Ossenkoppele, R. (2020). Amyloid-beta-independent regulators of tau pathology in Alzheimer disease. Nature Reviews Neuroscience, 21(1), 21–35. 10.1038/s41583-019-0240-3, 31780819

[bib47] Vazquez-Rodriguez, B., Suarez, L. E., Markello, R. D., Shafiei, G., Paquola, C., Hagmann, P., van den Heuvel, M. P., Bernhardt, B. C., Spreng, R. N., & Mišić, B. (2019). Gradients of structure-function tethering across neocortex. Proceedings of the National Academy of Sciences, 116(42), 21219–21227. 10.1073/pnas.1903403116, 31570622 PMC6800358

[bib48] Wang, H., Kulas, J. A., Wang, C., Holtzman, D. M., Ferris, H. A., & Hansen, S. B. (2021). Regulation of beta-amyloid production in neurons by astrocyte-derived cholesterol. Proceedings of the National Academy of Sciences, 118(33), e2102191118. 10.1073/pnas.2102191118, 34385305 PMC8379952

[bib49] Wang, W. Y., Yu, J. T., Liu, Y., Yin, R. H., Wang, H. F., Wang, J., Tan, L., Radua, J., & Tan, L. (2015). Voxel-based meta-analysis of grey matter changes in Alzheimer’s disease. Translational Neurodegeneration, 4, 6. 10.1186/s40035-015-0027-z, 25834730 PMC4381413

[bib50] Wang, X., Wang, M., Wang, X., Zhou, F., Jiang, J., Liu, H., & Han, Y. (2021). Subjective cognitive decline–related worries modulate the relationship between global amyloid load and gray matter volume in preclinical Alzheimer’s disease. Brain Imaging and Behavior, 16, 1088–1097. 10.1007/s11682-021-00558-w, 34743296

[bib51] Yang, J., Pan, P., Song, W., Huang, R., Li, J., Chen, K., Gong, Q., Zhong, J., Shi, H., & Shang, H. (2012). Voxelwise meta-analysis of gray matter anomalies in Alzheimer’s disease and mild cognitive impairment using anatomic likelihood estimation. Journal of the Neurological Sciences, 316(1–2), 21–29. 10.1016/j.jns.2012.02.010, 22385679

[bib52] Young, A. L., Marinescu, R. V., Oxtoby, N. P., Bocchetta, M., Yong, K., Firth, N. C., Cash, D. M., Thomas, D. L., Dick, K. M., Cardoso, J., van Swieten, J., Borroni, B., Galimberti, D., Masellis, M., Tartaglia, M. C., Rowe, J. B., Graff, C., Tagliavini, F., Frisoni, G. B., … Alzheimer’s Disease Neuroimaging Initiative. (2018). Uncovering the heterogeneity and temporal complexity of neurodegenerative diseases with Subtype and Stage Inference. Nature Communications, 9(1), 4273. 10.1038/s41467-018-05892-0, 30323170 PMC6189176

[bib53] Zhang, L., Mak, E., Reilhac, A., Shim, H. Y., Ng, K. K., Ong, M. Q. W., Ji, F., Chong, E. J. Y., Xu, X., Wong, Z. X., Stephenson, M. C., Venketasubramanian, N., Tan, B. Y., O’Brien, J. T., Zhou, J. H., Chen, C. L. H., & Alzheimer’s Disease Neuroimaging Initiative. (2020). Longitudinal trajectory of Amyloid-related hippocampal subfield atrophy in nondemented elderly. Human Brain Mapping, 41(8), 2037–2047. 10.1002/hbm.24928, 31944479 PMC7267893

[bib54] Zhao, K., Ding, Y., Han, Y., Fan, Y., Alexander-Bloch, A. F., Han, T., Jin, D., Liu, B., Lu, J., Song, C., Wang, P., Wang, D., Wang, Q., Xu, K., Yang, H., Yao, H., Zheng, Y., Yu, C., Zhou, B., … Liu, Y. (2020). Independent and reproducible hippocampal radiomic biomarkers for multisite Alzheimer’s disease: Diagnosis, longitudinal progress and biological basis. Science Bulletin, 65(13), 1103–1113. 10.1016/j.scib.2020.04.003 36659162

[bib55] Zhao, K., Zheng, Q., Che, T., Dyrba, M., Li, Q., Ding, Y., Zheng, Y., Liu, Y., & Li, S. (2021). Regional radiomics similarity networks (R2SNs) in the human brain: Reproducibility, small-world properties and a biological basis. Network Neuroscience, 5(3), 783–797. 10.1162/netn_a_00200, 34746627 PMC8567836

[bib56] Zhao, K., Zheng, Q., Dyrba, M., Rittman, T., Li, A., Che, T., Chen, P., Sun, Y., Kang, X., Li, Q., Liu, B., Liu, Y., Li, S., & Alzheimer’s Disease Neuroimaging Initiative. (2022). Regional radiomics similarity networks reveal distinct subtypes and abnormality patterns in mild cognitive impairment. Advanced Science, 9(12), e2104538. 10.1002/advs.202104538, 35098696 PMC9036024

