# Supplementary material for: Coupling of the spatial distributions between sMRI and PET reveals the progression of Alzheimer’s disease
Source: Netw Neurosci. 2023 Jan 1;7(1):86–101. doi: 10.1162/netn_a_00271 (PMC10270713; doi:10.1162/netn_a_00271)
Supplement: Supplementary file 1 [file netn-7-1-86-s001.pdf]

Zhao, K., Lin, J., Dyrba, M., Wang, D., Che, T., Wu, H., Wang, J., Liu, Y. & Li, S. (2023). Supporting information for "Coupling of the spatial distributions between sMRI and PET reveals the progression of Alzheimer's disease." *Network Neuroscience*, 7(1), 86–101. https://doi.org/10.1162/netn_a_00271

# Regional radiomics similarity networks reveal distinct subtypes and abnormality patterns in mild cognitive impairment

## S01: Data description

Additional information about the Alzheimer's Disease Neuroimaging Initiative (ADNI) dataset was found in http://adni.loni.usc.edu/wp-content/uploads/how_to_apply/ADNI_Acknowledgement_List.pdf. The clinical information of the baseline was used in this study (https://ida.loni.usc.edu/pages/access/studyData.jsp), and the detailed information about the clinical measures is shown in Table 1.

Data collection and sharing for this project were funded by the Alzheimer's Disease Neuroimaging Initiative (ADNI) (National Institutes of Health Grant U01 AG024904) and DOD ADNI (Department of Defense award number W81XWH-12-2-0012). ADNI is funded by the National Institute on Aging, the National Institute of Biomedical Imaging and Bioengineering, and generous contributions from AbbVie, Alzheimer's Association; Alzheimer's Drug Discovery Foundation; Araclon Biotech; BioClinica, Inc.; Biogen; Bristol-Myers Squibb Company; CereSpir, Inc.; Cogstate; Eisai Inc.; Elan Pharmaceuticals, Inc.; Eli Lilly and Company; EuroImmun; F. Hoffmann-La Roche Ltd, and its affiliated company Genentech, Inc.; Fujirebio; GE Healthcare; IXICO Ltd.; Janssen Alzheimer Immunotherapy Research & Development, LLC.; Johnson & Johnson Pharmaceutical Research & Development LLC.; Lumosity; Lundbeck; Merck & Co., Inc.; Meso Scale Diagnostics, LLC.; NeuroRx Research; Neurotrack Technologies; Novartis Pharmaceuticals Corporation; Pfizer Inc.; Piramal Imaging; Servier; Takeda Pharmaceutical Company; and Transition Therapeutics. The Canadian Institutes of Health Research provide funds to support ADNI clinical sites in Canada. Private sector contributions are facilitated by the Foundation for the National Institutes of Health (www.fnih.org). The grantee organization is the Northern California Institute for Research and Education, and the study is coordinated by the Alzheimer's Therapeutic Research Institute at the University of Southern California. The ADNI data are disseminated by the Laboratory for Neuro Imaging at the University of Southern California.

# S02: Definitions of the radiomics features

A total of 47 MRI imaging features, including intensity, textural features, were extracted in this study (Aerts et al., 2014). We provided this information in a previous study(Feng et al., 2018; Zhao et al., 2020). To maintain the integrity of this study, we also list it here.

## 1. Intensity features

**Table S2.** Intensity features describe the distribution of voxel intensities within an MRI image through commonly used and basic metrics. Because some intensity features have a close relationship with the voxel intensity, such as the maximum and mean, we first normalized those features by the image intensity using the min-max method in each center.

| | Image feature | Equation | Definition |
|---|---|---|---|
| Intensity features (14) | Energy | $\sum_{i}^{N} X(i)^2$ | Measure of the randomness of the intensity values in an image |
| | Entropy | $\sum_{i=1}^{N_l} P(i)\log_2 P(i)$ | Represents the irregularity of the intensity value distribution |
| | Kurtosis | $\frac{\frac{1}{N}\sum_{i=1}^{N}(X(i)-\overline{X})^4}{\left(\sqrt{\frac{1}{N}\sum_{i=1}^{N}(X(i)-\overline{X})^2}\right)^2}$ | The peakedness of the histogram or indication of histogram flatness |
| | Maximum | Maximum intensity value of X | |
| | Mean | $\frac{1}{N}\sum_{i}^{N} X(i)$ | Average intensity value of the pixels within the region of interest |
| | Mean Absolute Deviation (mad) | Mean of the absolute deviations of all voxel intensities around the mean intensity value | A measure of how much the gray levels differ from the mean |

| | | |
|---|---|---|
| Median | Median intensity value of X | |
| Minimum | Minimum intensity value of X | |
| Range | Range of intensity values of X | |
| Root Mean Square (RMS) | $\sqrt{\frac{\sum_{i}^{N} X(i)^2}{N}}$ | |
| Skewness | $\frac{\frac{1}{N}\sum_{i=1}^{N}(X(i)-\bar{X})^3}{\left(\sqrt{\frac{1}{N}\sum_{i=1}^{N}(X(i)-\bar{X})^2}\right)^3}$ | Symmetry of intensity values in an image |
| Standard Deviation | $\left(\frac{1}{N-1}\sum_{i=1}^{N}(X(i)-\bar{X})^2\right)^{1/2}$ | A measure of how much variation or dispersion exists |
| Uniformity | $\sum_{i=1}^{N_l} P(i)^2$ | Measures the homogeneity of the intensity value distribution in an image |
| Variance (Var) | $\frac{1}{N-1}\sum_{i=1}^{N}(X(i)-\bar{X})^2$ | The spread or variation around the mean (sum of squares) |

**X** denotes the three-dimensional image matrix. ***N*** is the number of voxels. **P** is the first-order histogram with $N_l$ discrete intensity levels. $\bar{X}$ is the mean of X. The number of histogram bins is 100.

## 2. Textural features

**Table S3.** Textural features describe the patterns or spatial distribution of voxel intensities.

| | Image feature | Equation | Definition |
|---|---|---|---|
| | Autocorrelation | $\sum_{i=1}^{N_g}\sum_{j=1}^{N_g} ij\mathrm{P}(i,j)$ | |
| | Cluster Prominence (CP) | $\sum_{i=1}^{N_g}\sum_{j=1}^{N_g}\left[i+j-\mu_x(i)-\mu_y(j)\right]^4\mathrm{P}(i,j)$ | |
| | Cluster Shade | $\sum_{i=1}^{N_g}\sum_{j=1}^{N_g}\left[i+j-\mu_x(i)-\mu_y(j)\right]^3\mathrm{P}(i,j)$ | |
| | Cluster Tendency | $\sum_{i=1}^{N_g}\sum_{j=1}^{N_g}\left[i+j-\mu_x(i)-\mu_y(j)\right]^2\mathrm{P}(i,j)$ | |
| | Contrast | $\sum_{i=1}^{N_g}\sum_{j=1}^{N_g}\lvert i-j\rvert^2\,\mathrm{P}(i,j)$ | Measures the local variation in intensity values |
| Textural features (33) | Correlation | $\frac{\sum_{i=1}^{N_g}\sum_{j=1}^{N_g} ijP(i,j)-\mu_i(i)\mu_j(j)}{\sigma_x(i)\sigma_y(j)}$ | Measures the linear dependencies of intensity values in an image |
| | Difference Entropy | $\sum_{i=0}^{N_g-1} P_{x-y}(i)log_2\left[P_{x-y}(i)\right]$ | |
| | Dissimilarity | $\sum_{i=1}^{N_g}\sum_{j=1}^{N_g}\lvert i-j\rvert\,\mathrm{P}(i,j)$ | |
| | Energy | $\sum_{i=1}^{N_g}\sum_{j=1}^{N_g}[\mathrm{P}(i,j)]^2$ | |
| | Entropy | $-\sum_{i=1}^{N_g}\sum_{j=1}^{N_g}\mathrm{P}(i,j)\,log_2[\mathrm{P}(i,j)]$ | |
| | Homogeneity1 | $\sum_{i=1}^{N_g}\sum_{j=1}^{N_g}\frac{\mathrm{P}(i,j)}{1+\lvert i-j\rvert}$ | Measures the homogeneity of the intensity values |

| | | |
|---|---|---|
| Homogeneity2 | $\sum_{i=1}^{N_g}\sum_{j=1}^{N_g}\frac{\mathrm{P}(i,j)}{1+\lvert i-j\rvert^2}$ | Measures the homogeneity of the intensity values of the pixel pair |
| Informational Measure of Correlation 1 (IMC1) | $\frac{HXY-HXY1}{max\{HX,HY\}}$ | |
| Informational Measure of Correlation 2 (IMC2) | $\sqrt{1-e^{-2(HXY2-HXY)}}$ | |
| Inverse Difference Moment Normalized (IDMN) | $\sum_{i=1}^{N_g}\sum_{j=1}^{N_g}\frac{\mathrm{P}(i,j)}{1+\left(\frac{\lvert i-j\rvert^2}{N^2}\right)}$ | |
| Inverse Difference Normalized (IDN) | $\sum_{i=1}^{N_g}\sum_{j=1}^{N_g}\frac{\mathrm{P}(i,j)}{1+\left(\frac{\lvert i-j\rvert}{N}\right)}$ | |
| Inverse Variance | $\sum_{i=1}^{N_g}\sum_{j=1}^{N_g}\frac{\mathrm{P}(i,j)}{\lvert i-j\rvert^2}, i\neq j$ | |
| Maximum Probability | $\max\{P(i,j)\}$ | |
| Sum Average | $\sum_{i=2}^{2N_g}\left[i\mathrm{P}_{x+y}(i)\right]$ | |
| Sum Entropy | $-\sum_{i=2}^{2N_g}\mathrm{P}_{x+y}(i)log_2\left[\mathrm{P}_{x+y}(i)\right]$ | |
| Sum Variance | $\sum_{i=2}^{2N_g}(i-SE)^2\,\mathrm{P}_{x+y}(i)$ | |
| Variance | $\sum_{i=1}^{N_g}\sum_{j=1}^{N_g}(i-\mu)^2\,\mathrm{P}(i,j)$ | |
| Short Run Emphasis (SRE) | $\frac{\sum_{i=1}^{N_g}\sum_{j=1}^{N_r}\left[\frac{p(i,j\lvert\theta)}{j^2}\right]}{\sum_{i=1}^{N_g}\sum_{j=1}^{N_r}p(i,j\lvert\theta)}$ | |
| Long Run Emphasis (LRE) | $\frac{\sum_{i=1}^{N_g}\sum_{j=1}^{N_r}j^2\,p(i,j\lvert\theta)}{\sum_{i=1}^{N_g}\sum_{j=1}^{N_r}p(i,j\lvert\theta)}$ | |
| Gray Level Nonuniformity (GLN) | $\frac{\sum_{i=1}^{N_g}\left[\sum_{j=1}^{N_r}p(i,j\lvert\theta)\right]^2}{\sum_{i=1}^{N_g}\sum_{j=1}^{N_r}p(i,j\lvert\theta)}$ | Represents the similarity of intensity values in an image |

$\mathrm{P(i,j)}$ is the co-occurrence matrix for an arbitrary $\delta$ and $\alpha$

$N_g$ is the number of discrete intensity levels in the image

$p(i,j|\theta)$ is the $(i,j)$th entry in the given run-length matrix $p$ for a direction $\theta$

$N_g$ is the number of discrete intensity values in the image

$N_r$ is the number of different run lengths

$N_p$ is the number of voxels in the image

u is the mean of $\mathrm{P(i,j)}$

$\mathrm{p_x(i)}$=$\sum_{\mathrm{j=1}}^{\mathrm{N_g}} \mathrm{P(i,j)}$ is the marginal row probabilities

$\mathrm{p_y(i)}$=$\sum_{\mathrm{i=1}}^{\mathrm{N_g}} \mathrm{P(i,j)}$ is the marginal column probabilities

$\mu_x$ is the mean of $p_x$

$\mu_y$ is the mean of $p_y$

$\sigma_x$ is the standard deviation of $p_x$

$\sigma_y$ is the standard deviation of $p_y$

$\mathrm{p_{x+y}(k)}$=$\sum_{\mathrm{i=1}}^{\mathrm{N_g}}\sum_{\mathrm{j=1}}^{\mathrm{N_g}} \mathrm{P(i,j)}$, i+j=k, k=2,3,…,2*$N_g$

$\mathrm{P_{x-y}(k)}$=$\sum_{\mathrm{i=1}}^{\mathrm{N_g}}\sum_{\mathrm{j=1}}^{\mathrm{N_g}} \mathrm{P(i,j)}$,$|\mathrm{i}-\mathrm{j}|$=k, k=0,1,…,$N_g$ −1

HX=$-\sum_{\mathrm{i=1}}^{\mathrm{N_g}} \mathrm{p_x}\,(i)\log_2[\mathrm{p_x(i)}]$ is the entropy of $\mathrm{p_x}$

HY=$-\sum_{\mathrm{i=1}}^{\mathrm{N_g}} \mathrm{p}_y\,(i)\log_2\left[\mathrm{p_y(i)}\right]$ is the entropy of $\mathrm{p_y}$

H=$-\sum_{\mathrm{i=1}}^{\mathrm{N_g}}\sum_{j=1}^{N_g} P(i,j)\log_2[P(i,j)]$ is the entropy of $P(i,j)$

HXY1=$-\sum_{i=1}^{N_g}\sum_{j=1}^{N_g} P(i,j)log\left(p_x(i)p_y(j)\right)$

HXY2=$-\sum_{i=1}^{N_g}\sum_{j=1}^{N_g} p_x(i)p_y(j)log\left(p_x(i)p_y(j)\right)$

## S03: Supplementary method for R2SN construction

We first performed a common min-max method to normalize the radiomics feature among different brain regions in an individual, and the redundancy feature was defined as those features which had a high correlation with others feature (R>0.9) (Zhao et al., 2021). Those superfluous features were removed before subsequent analysis. As a result, a final feature matrix with 25 × 246 (Brainnetome atlas (Fan et al., 2016)) for each subject was obtained for further analysis. The reserved features are shown in Table S4. The detailed brain regions' names of the Brainnetome atlas are shown in Table S5.

**Table S4.** The reserved features after removing superfluous features

| | |
|---|---|
| **Intensity features** | energy |
| | kurtosis |
| | maximum |
| | mad |
| | minimum |
| | skewness |
| | entropy |
| **Textural features** | Autocorrelation |
| | Cluster Prominence |
| | Cluster Shade |
| | Cluster Tendency |
| | Contrast |
| | Correlation |
| | Energy |
| | Entropy |
| | Homogeneity1 |
| | IMC1 |
| | Maximum Probability |
| | Sum Entropy |
| | Short Run Emphasis |
| | Long Run Emphasis |
| | Gray Level Nonuniformity |
| | Low Gray Level Run Emphasis |
| | High Gray Level Run Emphasis |
| | Long Run High Gray Level Emphasis |

**Table S5.** The detailed brain regions' name of Brainnetome atlas (https://atlas.brainnetome.org/).

| Lobe | Gyrus | Left and Right Hemisphere | Label ID.L | Label ID.R | Anatomical and modified Cyto-architectonic descriptions |
|---|---|---|---|---|---|
| Frontal Lobe | SFG, Superior Frontal Gyrus | SFG_L(R)_7_1 | 1 | 2 | *A8m, medial area 8* |
| | | SFG_L(R)_7_2 | 3 | 4 | *A8dl, dorsolateral area 8* |
| | | SFG_L(R)_7_3 | 5 | 6 | *A9l, lateral area 9* |
| | | SFG_L(R)_7_4 | 7 | 8 | *A6dl, dorsolateral area 6* |
| | | SFG_L(R)_7_5 | 9 | 10 | *A6m, medial area 6* |
| | | SFG_L(R)_7_6 | 11 | 12 | *A9m,medial area 9* |
| | | SFG_L(R)_7_7 | 13 | 14 | *A10m, medial area 10* |
| | MFG, Middle Frontal Gyrus | MFG_L(R)_7_1 | 15 | 16 | *A9/46d, dorsal area 9/46* |
| | | MFG_L(R)_7_2 | 17 | 18 | *IFJ, inferior frontal junction* |
| | | MFG_L(R)_7_3 | 19 | 20 | *A46, area 46* |
| | | MFG_L(R)_7_4 | 21 | 22 | *A9/46v, ventral area 9/46* |
| | | MFG_L(R)_7_5 | 23 | 24 | *A8vl, ventrolateral area 8* |
| | | MFG_L(R)_7_6 | 25 | 26 | *A6vl, ventrolateral area 6* |
| | | MFG_L(R)_7_7 | 27 | 28 | *A10l, lateral area10* |
| | IFG, Inferior Frontal Gyrus | IFG_L(R)_6_1 | 29 | 30 | *A44d,dorsal area 44* |
| | | IFG_L(R)_6_2 | 31 | 32 | *IFS, inferior frontal sulcus* |
| | | IFG_L(R)_6_3 | 33 | 34 | *A45c, caudal area 45* |
| | | IFG_L(R)_6_4 | 35 | 36 | *A45r, rostral area 45* |
| | | IFG_L(R)_6_5 | 37 | 38 | *A44op, opercular area 44* |
| | | IFG_L(R)_6_6 | 39 | 40 | *A44v, ventral area 44* |
| | OrG, Orbital Gyrus | OrG_L(R)_6_1 | 41 | 42 | *A14m, medial area 14* |
| | | OrG_L(R)_6_2 | 43 | 44 | *A12/47o, orbital area 12/47* |
| | | OrG_L(R)_6_3 | 45 | 46 | *A11l, lateral area 11* |
| | | OrG_L(R)_6_4 | 47 | 48 | *A11m, medial area 11* |
| | | OrG_L(R)_6_5 | 49 | 50 | *A13, area 13* |
| | | OrG_L(R)_6_6 | 51 | 52 | *A12/47l, lateral area 12/47* |
| | PrG, Precentral Gyrus | PrG_L(R)_6_1 | 53 | 54 | *A4hf, area 4(head and face region)* |
| | | PrG_L(R)_6_2 | 55 | 56 | *A6cdl, caudal dorsolateral area 6* |
| | | PrG_L(R)_6_3 | 57 | 58 | *A4ul, area 4(upper limb region)* |
| | | PrG_L(R)_6_4 | 59 | 60 | *A4t, area 4(trunk region)* |
| | | PrG_L(R)_6_5 | 61 | 62 | *A4tl, area 4(tongue and larynx region)* |
| | | PrG_L(R)_6_6 | 63 | 64 | *A6cvl, caudal ventrolateral area 6* |
| | PCL, Paracentral | PCL_L(R)_2_1 | 65 | 66 | *A1/2/3ll, area1/2/3 (lower limb* |

| | | | | | |
|---|---|---|---|---|---|
| | Lobule | | | | *region)* |
| | | PCL_L(R)_2_2 | 67 | 68 | *A4ll, area 4, (lower limb region)* |
| **Temporal Lobe** | STG, Superior Temporal Gyrus | STG_L(R)_6_1 | 69 | 70 | *A38m, medial area 38* |
| | | STG_L(R)_6_2 | 71 | 72 | *A41/42, area 41/42* |
| | | STG_L(R)_6_3 | 73 | 74 | *TE1.0 and TE1.2* |
| | | STG_L(R)_6_4 | 75 | 76 | *A22c, caudal area 22* |
| | | STG_L(R)_6_5 | 77 | 78 | *A38l, lateral area 38* |
| | | STG_L(R)_6_6 | 79 | 80 | *A22r, rostral area 22* |
| | MTG, Middle Temporal Gyrus | MTG_L(R)_4_1 | 81 | 82 | *A21c, caudal area 21* |
| | | MTG_L(R)_4_2 | 83 | 84 | *A21r, rostral area 21* |
| | | MTG_L(R)_4_3 | 85 | 86 | *A37dl, dorsolateral area37* |
| | | MTG_L(R)_4_4 | 87 | 88 | *aSTS, anterior superior temporal sulcus* |
| | ITG, Inferior Temporal Gyrus | ITG_L(R)_7_1 | 89 | 90 | *A20iv, intermediate ventral area 20* |
| | | ITG_L(R)_7_2 | 91 | 92 | *A37elv, extreme lateroventral area37* |
| | | ITG_L(R)_7_3 | 93 | 94 | *A20r, rostral area 20* |
| | | ITG_L(R)_7_4 | 95 | 96 | *A20il, intermediate lateral area 20* |
| | | ITG_L(R)_7_5 | 97 | 98 | *A37vl, ventrolateral area 37* |
| | | ITG_L(R)_7_6 | 99 | 100 | *A20cl, caudolateral of area 20* |
| | | ITG_L(R)_7_7 | 101 | 102 | *A20cv, caudoventral of area 20* |
| | FuG, Fusiform Gyrus | FuG_L(R)_3_1 | 103 | 104 | *A20rv, rostroventral area 20* |
| | | FuG_L(R)_3_2 | 105 | 106 | *A37mv, medioventral area37* |
| | | FuG_L(R)_3_3 | 107 | 108 | *A37lv, lateroventral area37* |
| | PhG, Parahippocampal Gyrus | PhG_L(R)_6_1 | 109 | 110 | *A35/36r, rostral area 35/36* |
| | | PhG_L(R)_6_2 | 111 | 112 | *A35/36c, caudal area 35/36* |
| | | PhG_L(R)_6_3 | 113 | 114 | *TL, area TL (lateral PPHC, posterior parahippocampal gyrus)* |
| | | PhG_L(R)_6_4 | 115 | 116 | *A28/34, area 28/34 (EC, entorhinal cortex)* |
| | | PhG_L(R)_6_5 | 117 | 118 | *TI, area TI(temporal agranular insular cortex)* |
| | | PhG_L(R)_6_6 | 119 | 120 | *TH, area TH (medial PPHC)* |
| | pSTS, posterior Superior Temporal Sulcus | pSTS_L(R)_2_1 | 121 | 122 | *rpSTS, rostroposterior superior temporal sulcus* |
| | | pSTS_L(R)_2_2 | 123 | 124 | *cpSTS, caudoposterior superior temporal sulcus* |
| **Parietal** | SPL, Superior | SPL_L(R)_5_1 | 125 | 126 | *A7r, rostral area 7* |

| | | | | | |
|---|---|---|---|---|---|
| **Lobe** | Parietal Lobule | SPL_L(R)_5_2 | 127 | 128 | *A7c, caudal area 7* |
| | | SPL_L(R)_5_3 | 129 | 130 | *A5l, lateral area 5* |
| | | SPL_L(R)_5_4 | 131 | 132 | *A7pc, postcentral area 7* |
| | | SPL_L(R)_5_5 | 133 | 134 | *A7ip, intraparietal area 7(hIP3)* |
| | IPL, Inferior Parietal Lobule | IPL_L(R)_6_1 | 135 | 136 | *A39c, caudal area 39(PGp)* |
| | | IPL_L(R)_6_2 | 137 | 138 | *A39rd, rostrodorsal area 39(Hip3)* |
| | | IPL_L(R)_6_3 | 139 | 140 | *A40rd, rostrodorsal area 40(PFt)* |
| | | IPL_L(R)_6_4 | 141 | 142 | *A40c, caudal area 40(PFm)* |
| | | IPL_L(R)_6_5 | 143 | 144 | *A39rv, rostroventral area 39(PGa)* |
| | | IPL_L(R)_6_6 | 145 | 146 | *A40rv, rostroventral area 40(PFop)* |
| | Pcun, Precuneus | PCun_L(R)_4_1 | 147 | 148 | *A7m, medial area 7(PEp)* |
| | | PCun_L(R)_4_2 | 149 | 150 | *A5m, medial area 5(PEm)* |
| | | PCun_L(R)_4_3 | 151 | 152 | *dmPOS, dorsomedial parietooccipital sulcus(PEr)* |
| | | PCun_L(R)_4_4 | 153 | 154 | *A31, area 31 (Lc1)* |
| | PoG, Postcentral Gyrus | PoG_L(R)_4_1 | 155 | 156 | *A1/2/3ulhf, area 1/2/3(upper limb, head and face region)* |
| | | PoG_L(R)_4_2 | 157 | 158 | *A1/2/3tonIa, area 1/2/3(tongue and larynx region)* |
| | | PoG_L(R)_4_3 | 159 | 160 | *A2, area 2* |
| | | PoG_L(R)_4_4 | 161 | 162 | *A1/2/3tru, area1/2/3(trunk region)* |
| **Insular Lobe** | INS, Insular Gyrus | INS_L(R)_6_1 | 163 | 164 | *G, hypergranular insula* |
| | | INS_L(R)_6_2 | 165 | 166 | *vIa, ventral agranular insula* |
| | | INS_L(R)_6_3 | 167 | 168 | *dIa, dorsal agranular insula* |
| | | INS_L(R)_6_4 | 169 | 170 | *vId/vIg, ventral dysgranular and granular insula* |
| | | INS_L(R)_6_5 | 171 | 172 | *dIg, dorsal granular insula* |
| | | INS_L(R)_6_6 | 173 | 174 | *dId, dorsal dysgranular insula* |
| **Limbic Lobe** | CG, Cingulate Gyrus | CG_L(R)_7_1 | 175 | 176 | *A23d, dorsal area 23* |
| | | CG_L(R)_7_2 | 177 | 178 | *A24rv, rostroventral area 24* |
| | | CG_L(R)_7_3 | 179 | 180 | *A32p, pregenual area 32* |
| | | CG_L(R)_7_4 | 181 | 182 | *A23v, ventral area 23* |
| | | CG_L(R)_7_5 | 183 | 184 | *A24cd, caudodorsal area 24* |
| | | CG_L(R)_7_6 | 185 | 186 | *A23c, caudal area 23* |
| | | CG_L(R)_7_7 | 187 | 188 | *A32sg, subgenual area 32* |
| **Occipital Lobe** | MVOcC, MedioVentral | MVOcC _L(R)_5_1 | 189 | 190 | *cLinG, caudal lingual gyrus* |
| | | MVOcC _L(R)_5_2 | 191 | 192 | *rCunG, rostral cuneus gyrus* |

| | | | | | |
|---|---|---|---|---|---|
| | Occipital Cortex | MVOcC _L(R)_5_3 | 193 | 194 | *cCunG, caudal cuneus gyrus* |
| | | MVOcC _L(R)_5_4 | 195 | 196 | *rLinG, rostral lingual gyrus* |
| | | MVOcC _L(R)_5_5 | 197 | 198 | *vmPOS,ventromedial parietooccipital sulcus* |
| | LOcC, lateral Occipital Cortex | LOcC_L(R)_4_1 | 199 | 200 | *mOccG, middle occipital gyrus* |
| | | LOcC _L(R)_4_2 | 201 | 202 | *V5/MT+, area V5/MT+* |
| | | LOcC _L(R)_4_3 | 203 | 204 | *OPC, occipital polar cortex* |
| | | LOcC_L(R)_4_4 | 205 | 206 | *iOccG, inferior occipital gyrus* |
| | | LOcC _L(R)_2_1 | 207 | 208 | *msOccG, medial superior occipital gyrus* |
| | | LOcC _L(R)_2_2 | 209 | 210 | *lsOccG, lateral superior occipital gyrus* |
| **Subcortical Nuclei** | Amyg, Amygdala | Amyg_L(R)_2_1 | 211 | 212 | *mAmyg, medial amygdala* |
| | | Amyg_L(R)_2_2 | 213 | 214 | *lAmyg, lateral amygdala* |
| | Hipp, Hippocampus | Hipp_L(R)_2_1 | 215 | 216 | *rHipp, rostral hippocampus* |
| | | Hipp_L(R)_2_2 | 217 | 218 | *cHipp, caudal hippocampus* |
| | BG, Basal Ganglia | BG_L(R)_6_1 | 219 | 220 | *vCa, ventral caudate* |
| | | BG_L(R)_6_2 | 221 | 222 | *GP, globus pallidus* |
| | | BG_L(R)_6_3 | 223 | 224 | *NAC, nucleus accumbens* |
| | | BG_L(R)_6_4 | 225 | 226 | *vmPu, ventromedial putamen* |
| | | BG_L(R)_6_5 | 227 | 228 | *dCa, dorsal caudate* |
| | | BG_L(R)_6_6 | 229 | 230 | *dlPu, dorsolateral putamen* |
| | Tha, Thalamus | Tha_L(R)_8_1 | 231 | 232 | *mPFtha, medial pre-frontal thalamus* |
| | | Tha_L(R)_8_2 | 233 | 234 | *mPMtha, pre-motor thalamus* |
| | | Tha_L(R)_8_3 | 235 | 236 | *Stha, sensory thalamus* |
| | | Tha_L(R)_8_4 | 237 | 238 | *rTtha, rostral temporal thalamus* |
| | | Tha_L(R)_8_5 | 239 | 240 | *PPtha, posterior parietal thalamus* |
| | | Tha_L(R)_8_6 | 241 | 242 | *Otha, occipital thalamus* |
| | | Tha_L(R)_8_7 | 243 | 244 | *cTtha, caudal temporal thalamus* |
| | | Tha_L(R)_8_8 | 245 | 246 | *lPFtha, lateral pre-frontal thalamus* |

## S04. Supplementary results

**Table S6.** Detailed correlation results between coupling score and clinical measures with the different covariates.

| | Correlation analysis with age and sex as a covariate | | Correlation analysis with age, sex, and education as a covariate | |
|---|---|---|---|---|
| | **R value** | **P value** | **R value** | **P value** |
| MMSE | 0.2207 | <0.001 | 0.2160 | <0.001 |
| AVLT1 | 0.2774 | <0.001 | 0.2700 | <0.001 |
| AVLT2 | 0.2372 | <0.001 | 0.2332 | <0.001 |
| ADAS-Cog11 | -0.2734 | <0.001 | -0.2696 | <0.001 |
| ADAS-Cog13 | -0.2930 | <0.001 | -0.2890 | <0.001 |
| Abeta | 0.4052 | <0.001 | 0.4020 | <0.001 |
| FDG | 0.2861 | <0.001 | 0.2831 | <0.001 |
| Tau | -0.3659 | <0.001 | -0.3643 | <0.001 |
| P-tau | -0.3879 | <0.001 | -0.3863 | <0.001 |

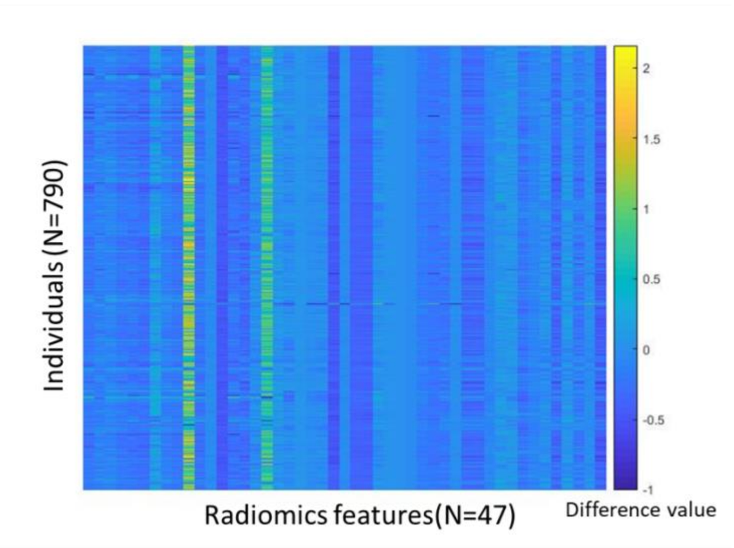


**Figure S1.** Difference of normalized radiomics features in ROI1 between sMRI and PET.

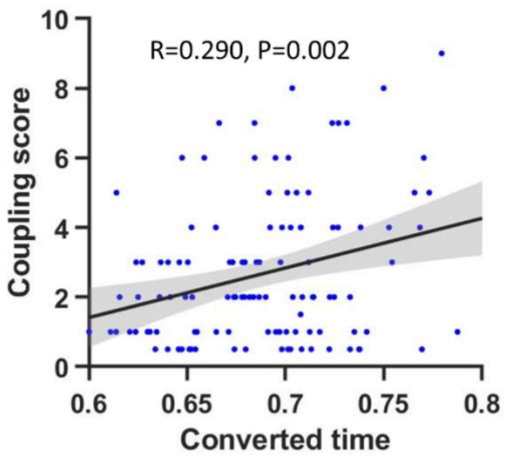


**Figure S2.** The correlation result between coupling score and converting time to the AD of pMCI individuals.

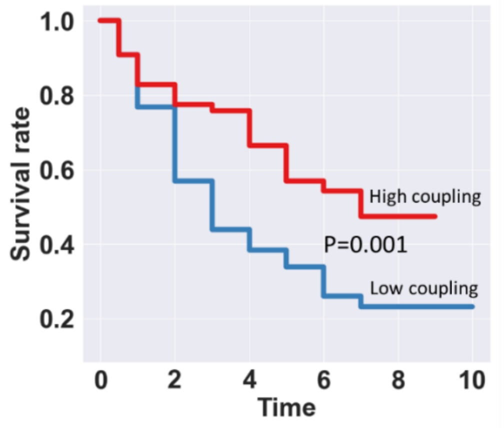


**Figure S3.** The survival curve of the different coupling levels in MCI with Aβ+.

# Reference

Aerts HJ, Velazquez ER, Leijenaar RT, Parmar C, Grossmann P, Carvalho S*, et al.* Decoding tumour phenotype by noninvasive imaging using a quantitative radiomics approach. Nat Commun 2014; 5: 4006.

Fan L, Li H, Zhuo J, Zhang Y, Wang J, Chen L*, et al.* The Human Brainnetome Atlas: A New Brain Atlas Based on Connectional Architecture. Cereb Cortex 2016; 26(8): 3508-26.

Feng F, Wang P, Zhao K, Zhou B, Yao H, Meng Q*, et al.* Radiomic Features of Hippocampal Subregions in Alzheimer's Disease and Amnestic Mild Cognitive Impairment. Front Aging Neurosci 2018; 10: 290.

Zhao K, Ding YH, Han Y, Fan Y, Alexander-Bloch AF, Han T*, et al.* Independent and reproducible hippocampal radiomic biomarkers for multisite Alzheimer's disease: diagnosis, longitudinal progress and biological basis. Science Bulletin 2020; 65(13): 1103-13.

Zhao K, Zheng Q, Che T, Dyrba M, Li Q, Ding Y*, et al.* Regional radiomics similarity networks (R2SNs) in the human brain: reproducibility, small-world properties and a biological basis. Network Neuroscience 2021: 1-30.
